# Melatonin Regulates Sheep Granulosa Cell Proliferation and Apoptosis Through a Novel Micropeptide, IGF1Raa

**DOI:** 10.3390/cells15191771

**Published:** 2026-09-29

**Authors:** Weiming Zhong, Jianyu Ma, Zhibo Wang, Hua Yang, Xiuyuan Wang, Yang Gao, Shang Gao, Peiyong Chen, Yongjie Wan, Yanli Zhang, Feng Wang

**Affiliations:** 1 Sanya Institute of Nanjing Agricultural University, Sanya 572024, China; 2 Jiangsu Livestock Embryo Engineering Laboratory, College of Animal Science and Technology, Nanjing Agricultural University, Nanjing 210095, China; 3 Shanghai Animal Disease Prevention and Control Center, Shanghai 201103, China

**Keywords:** GCs, micropeptide, sORF, melatonin, PP2A/AMPK/mTOR

## Abstract

**Highlights:**

**What are the main findings?**

- A 32-amino-acid micropeptide, IGF1Raa, encoded by a sORF within the 5′UTR of IGF1R, was identified.
- Melatonin downregulates the expression of this micropeptide, thereby modulating the PP2A/AMPK/mTOR signaling pathway and subsequently affecting the proliferation, apoptosis, and estrogen synthesis of sheep GCs.
- The function of IGF1Raa is independent of the CDS region of IGF1R, and no reciprocal regulation exists between them.

**What is the implication of the main finding?**

- Melatonin regulates granulosa cell function through a 5′UTR-encoded micropeptide, revealing a novel non-coding-region-dependent mechanism that expands the understanding of ovarian physiology beyond classical signaling pathways.
- The independence of IGF1Raa from the canonical IGF1R coding sequence suggests a distinct regulatory layer, positioning IGF1Raa as a potential therapeutic target for reproductive disorders without interfering with conventional IGF1R signaling.

**Abstract:**

Melatonin is a hormone primarily secreted by the pineal gland, best known for regulating circadian rhythms. It also plays a critical role in ovarian physiology by modulating granulosa cell (GC) function during follicular development, although the underlying molecular mechanisms remain incompletely understood. Recent advances in ribosome sequencing (Ribo-seq) have revealed that many small open reading frames (sORFs), previously overlooked in conventional annotations, actually encode functional micropeptides. However, whether melatonin exerts its ovarian effects through such micropeptides has remained unexplored. In the present study, we identified a previously unannotated sORF within the 5′untranslated region (UTR) of sheep IGF1R mRNA and demonstrated that this sORF encodes a 32-amino-acid micropeptide, which we named IGF1Raa. Knockdown and overexpression of IGF1Raa in sheep GCs, assessed by CCK-8, Edu, flow cytometry, and ELISA, revealed that IGF1Raa negatively regulates GC proliferation and E2 secretion and promotes apoptosis. Co-ip assays and RNA-seq show that IGF1Raa physically associates with PPP2R2B, a regulatory subunit of protein phosphatase 2A (PP2A), and modulates PP2A protein expression. Consequently, IGF1Raa regulates the phosphorylation of AMPK (Thr172) and its downstream effector Raptor (Ser792), thereby controlling the AMPK/mTOR signaling cascade. Importantly, melatonin suppresses IGF1Raa expression, and overexpression of IGF1Raa partially abrogates melatonin’s stimulatory effects on GC proliferation and steroidogenesis. Collectively, our findings identify IGF1Raa as a melatonin-responsive micropeptide that governs GC function through the PP2A/AMPK/mTOR axis, providing new insights into the regulatory network of ovarian physiology and offering potential therapeutic targets for reproductive disorders.

## 1. Introduction

The ovary is a dynamic endocrine organ whose function is tightly governed by the precise regulation of GC proliferation, apoptosis, and steroidogenesis [1]. As the primary somatic cells within ovarian follicles, GCs not only provide structural and nutritional support for oocyte development but also synthesize estradiol (E2) [2,3]. Increasing evidence indicates that the efficiency of GC function is a critical determinant of fertility and litter size in domestic ruminants, including the Hu sheep—a highly prolific breed renowned for its exceptional fecundity [4]. Despite extensive studies on the transcriptional and post-transcriptional regulation of GC biology [5], the molecular mechanisms that fine-tune GC fate decisions remain incompletely understood.

In mammals, most melatonin is secreted by the pineal gland [6]. Studies have reported that, in addition to the pineal gland, certain organs such as the ovary can also produce small amounts of melatonin [7]. Besides regulating circadian rhythms, melatonin is also involved in the control of animal reproductive activities [8]. In females, when the retina receives light signals, these signals are transmitted via neural pathways to the pineal gland, modulating melatonin secretion [9]. On one hand, melatonin secreted by the pineal gland acts directly on the hypothalamus to influence gonadotropin-releasing hormone (GnRH) secretion, thereby affecting the pituitary gland and gonads, and regulating estrus and reproduction [10]. On the other hand, melatonin can reach the ovary directly through the bloodstream and enter the follicles, where it not only acts as an antioxidant to scavenge excessive reactive oxygen species (ROS) [11] but also modulates GC function and influences E2 secretion [12]. To date, numerous studies have investigated the mechanisms by which melatonin regulates GCs. For example, melatonin promotes mitophagy via the SIRT1/FoxO1 signaling pathway, thereby attenuating oxidative stress-induced apoptosis and mitochondrial damage in bovine ovarian GCs [13]. It also alleviates oxidative stress in sheep GCs through the MAP3K8 and FOS pathways [14]. Additionally, melatonin regulates hormone secretion in porcine GCs via its membrane receptor MTNR1b [15]. However, whether melatonin regulates GC function through micropeptides remains largely unexplored.

Micropeptides are a class of short peptides, typically consisting of fewer than 100 amino acids and encoded by sORFs, which were frequently overlooked in conventional biological annotations [16]. With the recent advancement of Ribo-Seq technology, an increasing number of micropeptides have been reported to perform essential biological functions [17]. Examples include Minion, a micropeptide that promotes myoblast fusion [18], and MLN, a micropeptide that regulates cardiac muscle contraction [19]. In the field of reproduction, numerous micropeptides have also been recently characterized. Yu et al. demonstrated that GRN-122aa, encoded by lncGRN, participates in chicken follicular atresia by regulating GC proliferation, apoptosis, and hormone synthesis [20]. Shu et al. showed that HAND2OT-66aa, encoded by lncRNA HAND2OT, regulates E2 secretion, proliferation, and apoptosis in chicken ovarian theca cells [21]. Beyond ovarian function, micropeptides have been implicated in male reproductive regulation. Immarigeon et al. identified MSAmiP, a micropeptide specifically expressed in secondary cells of the Drosophila male accessory gland, and loss of this micropeptide resulted in defects in sperm competition [22]. In our previous study on melatonin and sheep GCs, we discovered a micropeptide, Nrf2aa, encoded by a sORF located in the 5′UTR of the Nrf2 mRNA. This micropeptide is regulated by melatonin and inversely modulates the translation efficiency of the Nrf2 CDS, thereby controlling the Nrf2/KEAP1 antioxidant pathway and ultimately affecting the antioxidant function of GCs [23]. However, in addition to Nrf2aa, we have identified other melatonin-responsive micropeptides in sheep GCs. The biological functions of these micropeptides and the mechanisms by which they are regulated by melatonin remain unclear and warrant further investigation.

In the present study, we focused on another previously identified melatonin-responsive micropeptide, IGF1Raa, which is encoded by a sORF located in the 5′UTR of the IGF1R mRNA. We investigated its coding capacity, biological functions in sheep GCs, and the underlying mechanisms. We confirmed that IGF1Raa is indeed translated in GCs and is regulated by melatonin. Furthermore, we examined the effects of IGF1Raa on GC proliferation, apoptosis, and E2 secretion. Notably, unlike Nrf2aa, which functions by modulating the translation efficiency of the Nrf2 CDS, IGF1Raa exerts its effects on GC function independently of IGF1R. Mechanistically, IGF1Raa physically interacts with PPP2R2B, a regulatory subunit of PP2A, resulting in decreased PP2A protein abundance, sustained phosphorylation of AMPK and Raptor, and subsequent activation of the AMPK/mTOR signaling cascade. Conversely, melatonin-mediated suppression of IGF1Raa relieves this inhibition, upregulates PP2A, inactivates AMPK/mTOR, and ultimately enhances GC viability and steroidogenesis. Our findings uncover a previously unrecognized melatonin-IGF1RaaPP2A-AMPK axis that orchestrates GC fate, providing new insights into the molecular architecture of ovarian follicular development and identifying a novel regulatory layer through which melatonin optimizes female reproductive function.

## 2. Materials and Methods

All experimental designs and operational procedures were reviewed and approved by the Institutional Animal Care and Use Committee of Nanjing Agricultural University, China, and were carried out in accordance with the approved guidelines.

### 2.1. Cell Culture

To dissect the cellular mechanisms underlying the in vivo observations, primary ovine granulosa cells (GCs) were carefully isolated from ovarian follicular fluid obtained from a local abattoir in Taicang, Jiangsu, China. Following isolation, the harvested GCs were plated onto 6-well culture plates and maintained in DMEM/F12 medium (Gibco, Thermo Fisher Scientific, Waltham, MA, USA) supplemented with 10% fetal bovine serum (FBS) and 1% penicillin-streptomycin (P/S). The cultures were incubated at 37 °C in a humidified atmosphere containing 5% CO_2_, with the culture medium being replaced every 24 h. Sheep ovarian GCs were isolated from ovaries collected at a slaughterhouse in Taicang, Jiangsu Province, China. Briefly, ovaries were collected after slaughter, placed in physiological saline pre-warmed to 37 °C, and immediately transported to the laboratory. Follicular fluid was aspirated from the ovaries and centrifuged at 1500 rpm for 10 min. The pellet was collected and washed twice with Dulbecco’s phosphate-buffered saline (DPBS). Subsequently, the pellet was resuspended in DMEM/F12 culture medium supplemented with 10% fetal bovine serum (FBS) and 1% penicillin-streptomycin, and seeded into 6-well plates. The cells were cultured at 37 °C in a humidified atmosphere containing 5% CO_2_. After 24 h, the culture medium was replaced to purify the cells. The obtained cells were used for subsequent experiments.

### 2.2. Transfection of siRNA and Overexpression Vectors

GCs were seeded into 6-well plates. When the cell density reached 50–60%, transfection of siRNA and overexpression plasmids was performed using Lipofectamine 3000 (Thermo Fisher Scientific, Waltham, MA, USA). Briefly, 5 μL of Lipofectamine 3000 was gently mixed with 125 μL of Opti-MEM medium (Gibco, Thermo Fisher Scientific, Waltham, MA, USA), while 5 μg of siRNA or overexpression vector was diluted in 125 μL of Opti-MEM medium. The diluted Lipofectamine 3000 and the diluted nucleic acids were then combined at a 1:1 ratio and incubated at room temperature for 15 min. The mixture was subsequently added to the GCs. After 6 h of incubation, the medium was replaced with complete culture medium.

### 2.3. Cell Treatment

At 24 h post-transfection, the culture medium was replaced, and the GCs were treated with melatonin or doxorubicin (DOX). Melatonin and DOX were dissolved in DMSO to prepare stock solutions at concentrations of 10 μM and 150 μM, respectively, and then diluted with complete culture medium to final working concentrations of 10 nM and 150 nM, respectively. The melatonin concentration was selected based on our previous study [23], while the DOX concentration was adopted from published report [24].

### 2.4. Cell Viability Assay

Subsequent to the treatments, cell viability was assessed using the Cell Counting Kit-8 (CCK-8; Vazyme, Nanjing, China). GCs were detached via trypsinization, centrifuged, resuspended, and seeded into 96-well plates. After incubation at 37 °C in a 5% CO_2_ humidified atmosphere for 4 h to allow attachment, the CCK-8 was added to each well, and the plates were further incubated at 37 °C for 1 h. The optical density was subsequently recorded at 450 nm using a spectrophotometer to determine cell viability.

### 2.5. RNA Extraction

At 36 h post-transfection and 12 h after melatonin and doxorubicin (DOX) treatment, total RNA was extracted from the cells using the TRIzol reagent (Takara, Dalian, China). Briefly, 1 mL of TRIzol reagent was added to the GCs in each well of a 6-well plate. The lysate was homogenized by repeated pipetting and transferred to a 1.5 mL microcentrifuge tube. Then, 200 μL of chloroform was added, and the tube was vigorously shaken until the mixture turned milky white. After incubation at room temperature for 5 min, the sample was centrifuged at 12,000× *g* for 15 min at 4 °C. The upper aqueous phase was carefully transferred to a new tube, and an equal volume of isopropanol was added. The tube was gently inverted to mix and incubated at room temperature for 10 min, followed by centrifugation at 12,000× *g* for 10 min at 4 °C. The supernatant was discarded. The RNA pellet was washed with 1 mL of pre-chilled 75% ethanol and centrifuged at 12,000× *g* for 5 min at 4 °C. After removing the supernatant, the pellet was air-dried at room temperature for 8–10 min. Finally, the RNA was dissolved in 20 μL of RNase-free water by gentle pipetting. The RNA concentration was measured using a UV spectrophotometer, and the RNA was used for subsequent experiments.

### 2.6. Quantitative Real-Time PCR (qRT-PCR)

Total RNA was adjusted to a concentration of 1 μg/μL, and 1 μg of total RNA was reverse transcribed into cDNA using the reverse transcription kit (Takara, Dalian, China) according to the manufacturer’s instructions.

The qPCR reaction system was prepared according to the manufacturer’s instructions (Vazyme, Nanjing, China), containing 1 μg of cDNA, 10 μL of SYBR Green master mix, 0.6 μL each of forward and reverse primers (10 μmol/L), and RNase-free water to a final volume of 20 μL. The mixture was thoroughly mixed and subjected to amplification on a real-time PCR system (Life Technologies, Waltham, MA, USA). The qPCR results were analyzed using the 2^−ΔΔCt^ method. The primer sequences used are listed in Table S1.

### 2.7. Co-Immunoprecipitation (Co-IP)

To identify potential protein–protein interactions, Co-IP assays were conducted. Cell lysates were collected and maintained at 4 °C to ensure complete lysis, followed by centrifugation at 12,000 rpm for 10 min. The resulting supernatants were carefully harvested; a small aliquot of each lysate was reserved for input controls, while the remainder was incubated with 10 μg of specific primary antibodies overnight at 4 °C. Pierce™ Protein A/G Agarose Beads (Thermo Fisher Scientific, Waltham, MA, USA) were pre-washed extensively with lysis buffer and then added to the lysates for immunoprecipitation overnight at 4 °C with gentle rotation. The immunocomplexes were pelleted by centrifugation at 2500 rpm for 3 min at 4 °C. After removing the supernatant, the beads were rinsed three times with 1 mL of lysis buffer. Finally, 100 μL of 2 × SDS sample loading buffer was added, and the samples were denatured by heating at 95 °C for 5 min, after which they were subjected to Western blotting (WB) and mass spectrometry (MS) analysis. Protein identification by MS was conducted by Mabnus Biotech Co., Inc. (Wuhan, China).

### 2.8. Western Blotting

To quantify protein expression levels, Western blotting was performed. Total cellular proteins were extracted with RIPA lysis buffer supplemented with PMSF (Thermo Fisher Scientific, Waltham, MA, USA) after 12 h of treatment. Protein concentrations were determined, and equal amounts of protein were separated by sodium dodecyl sulfate-polyacrylamide gel electrophoresis (SDS-PAGE). The resolved proteins were then electrotransferred onto polyvinylidene difluoride (PVDF) membranes (Millipore, Burlington, MA, USA). The membranes were blocked with 5% (*w*/*v*) non-fat dry milk in Tris-buffered saline for 1 h at room temperature, followed by overnight incubation with specific primary antibodies at 4 °C (antibodies listed in Supplementary Table S2). After three washes with Tris-buffered saline containing 0.1% Tween 20 (TBST), the membranes were incubated with horseradish peroxidase-conjugated secondary antibodies for 2 h at room temperature. Immunoreactive bands were visualized using an ECL system (LAS-3000, Fujifilm, Tokyo, Japan), and densitometric analysis was performed using ImageJ software (Version 1.54p, National Institutes of Health, Bethesda, MD, USA).

### 2.9. Edu Assay

In addition to overall viability, cell proliferation was specifically evaluated using an Edu staining assay (Beyotime, Shanghai, China) as previously described [23]. Briefly, cells were seeded in 12-well culture plates and incubated for 24 h, followed by melatonin treatment for the indicated durations. The medium was then replaced with complete medium containing 50 mmol/L Edu. After a 4 h incubation, the cells were washed three times with DPBS and fixed with 4% paraformaldehyde (PFA) at room temperature for 30 min. Next, the cells were treated with 2 mg/mL glycine for 5 min and permeabilized with DPBS containing 0.5% Triton X-100 for approximately 20 min. After additional washes with DPBS, the nuclei were counterstained with Hoechst 33342 at room temperature for 5 min. Finally, the stained cells were visualized and analyzed under a fluorescence microscope.

### 2.10. Flow Cytometry Analysis of Apoptosis

In parallel with proliferation assessment, apoptotic rates were determined using an Annexin V-FITC/PI detection kit (Universal, Shanghai, China) following the manufacturer’s protocol. After treatment, GCs were harvested, washed with DPBS, resuspended in binding buffer, and sequentially incubated with Annexin V-FITC and propidium iodide (PI) for 20 min in a light-protected environment. Subsequently, the apoptosis rate of GCs was detected using flow cytometry.

### 2.11. Flow Cytometry Analysis of Cell Cycle

To further characterize the cell population dynamics, cell cycle distribution was analyzed by flow cytometry using a PI staining kit (Xunbei, Nanjing, China) according to the manufacturer’s instructions. Following treatment, the harvested granulosa cells were washed with DPBS and fixed in 70% ethanol overnight at 4 °C. The fixed cells were then washed again, incubated with RNase A at 37 °C for 30 min, and stained with PI for 15 min in the dark. DNA content was measured using a flow cytometer, and the percentages of cells in the G0/G1, S, and G2/M phases were calculated.

### 2.12. ELISA Assay

To measure the concentration of E2 in culture medium, ELISA was performed. After 48 h of transfection followed by 12 h of melatonin exposure, both the culture supernatants were collected. The concentrations of E2 in the supernatants were determined using specific ELISA kits (Mairuida, Beijing, China), strictly in accordance with the manufacturer’s instructions.

### 2.13. RNA-Sequencing (RNA-Seq)

To comprehensively investigate the effect of IGF1Raa on the transcriptome of sheep GCs, RNA-seq was performed on GCs overexpressing IGF1Raa. The cDNA libraries were constructed using the NEB Next Ultra Directional RNA Library Prep Kit (New England Biolabs, Ipswich, MA, USA) and sequenced on the Illumina HiSeq (v2.2.58) X.10 platform at Gene Denovo Biotechnology Co., Ltd. (Guangzhou, China). After sequencing, raw reads underwent stringent quality control to remove low-quality sequences, yielding clean reads, which were subsequently aligned to the ovine reference genome (Oar_v4.0) using TopHat2. This alignment enabled the successful annotation of protein-coding genes. The abundance of gene transcripts was estimated by calculating fragments per kilobase of transcript per million mapped reads (FPKM) values using Cuffdiff software (version 2.1.1).

### 2.14. Statistical Analysis

All statistical analyses were conducted using SPSS Statistics for Windows (version 27.0; IBM Corp., Armonk, NY, USA). Comparisons between two groups were performed using Student’s *t*-test, while multiple group comparisons were evaluated by one-way analysis of variance (ANOVA) followed by appropriate post hoc tests, or by the non-parametric Kruskal–Wallis test where applicable. Each experiment was carried out in triplicate, and all data are expressed as the mean ± standard error of the mean (SEM). Differences were considered statistically significant at *p* < 0.05, * *p* < 0.01, and ** *p* < 0.001.

## 3. Results

### 3.1. Identification and Validation of IGF1Raa 

**Figure 1:**
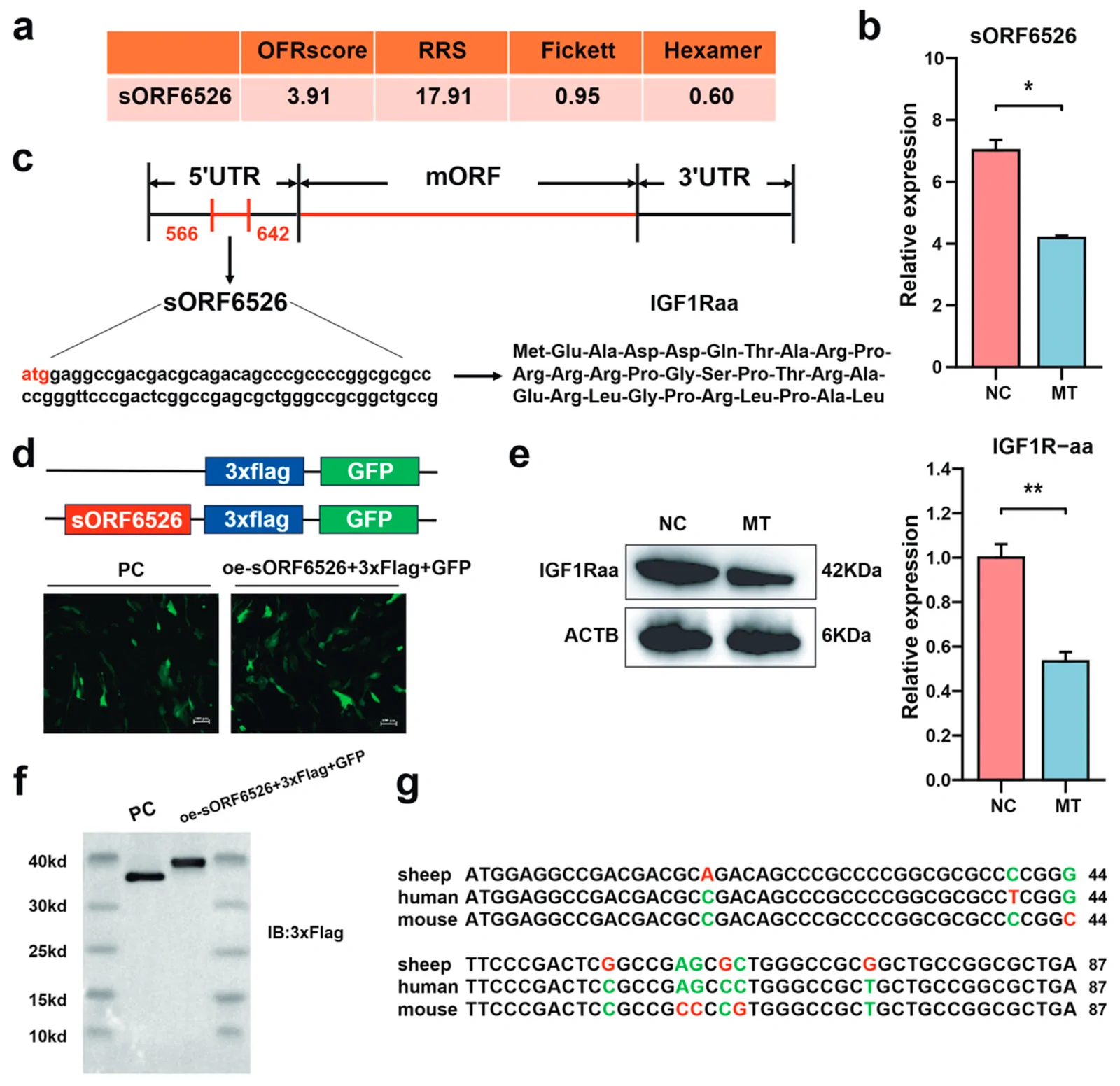
Identification and validation of IGF1Raa. (**a**) The coding potential scores of sORF6525 predicted by four software tools: OFRscore, RRS, Fickett, and Hexamer. (**b**) Comparison of sORF6526 expression levels before and after melatonin treatment. (**c**) Position of sORF6526 within the IGF1R mRNA, its nucleotide length, and the predicted 32-amino-acid peptide sequence. (**d**) Fluorescence microscopy of granulosa cells transfected with oe-sORF6526 (sORF6526 + 3 × Flag + GFP) or oe-NC (3 × Flag + GFP), showing green fluorescence indicating that the sORF is translated. (**e**) Comparison of IGF1Raa protein expression levels before and after melatonin treatment. (**f**) Western blot analysis of fusion protein expression after overexpression of the sORF. (**g**) Sequence homology alignment of corresponding sORFs in the 5′UTR of human, mouse, and sheep IGF1R mRNAs. NC denotes sheep GCs treated with 0.1% DMSO, and MT denotes GCs treated with melatonin (10 nM). * indicates *p* < 0.05, ** indicates *p* < 0.01 (n = 3).

In our previous study, through integrative analysis of Ribo-Seq and RNA-Seq data from melatonin-treated sheep GCs, we identified several sORFs with coding potential [23]. The present study focuses on sORF6526, located in the 5′UTR of the IGF1R mRNA, aiming to investigate its coding capacity and the biological functions and mechanisms of the micropeptide it encodes. Bioinformatics prediction indicated that sORF6526 possesses coding potential (Figure 1a), and Ribo-Seq data showed that melatonin treatment significantly reduced its expression level (Figure 1b). This sORF is situated 1817 nucleotides upstream of the canonical translation start site, consists of 87 nucleotides, and is predicted to encode a 32-amino-acid peptide with an estimated molecular mass of approximately 6 kDa (Figure 1c).

To experimentally validate the coding capacity of sORF6526, we constructed an overexpression vector harboring sORF6526 fused in-frame with a 3 × Flag tag and GFP (oe-sORF6526). Fluorescence microscopy of transfected GCs demonstrated that oe-sORF6526 produced green fluorescence comparable to that of the positive control (oe-NC, expressing GFP alone) (Figure 1d), confirming that sORF6526 is indeed translated into a peptide product. Immunoblotting further revealed that the oe-sORF6526 lysate exhibited a protein band slightly larger than that of oe-NC, consistent with the presence of the Flag-tagged peptide (Figure 1f). To detect IGF1Raa, a specific antibody was customized. The protein was confirmed to be expressed and, interestingly, its levels in granulosa cells were markedly reduced following melatonin treatment (Figure 1e). Collectively, these data establish that sORF6526 encodes a bona fide micropeptide, which we have termed IGF1Raa. Notably, we found that similar sORFs also exist in the 5′UTR of human and mouse IGF1R mRNAs, exhibiting high sequence homology with the ovine counterpart (Figure 1g).

### 3.2. Effects of IGF1Raa Knockdown on GC Proliferation, Apoptosis, and E2 Secretion

**Figure 2:**
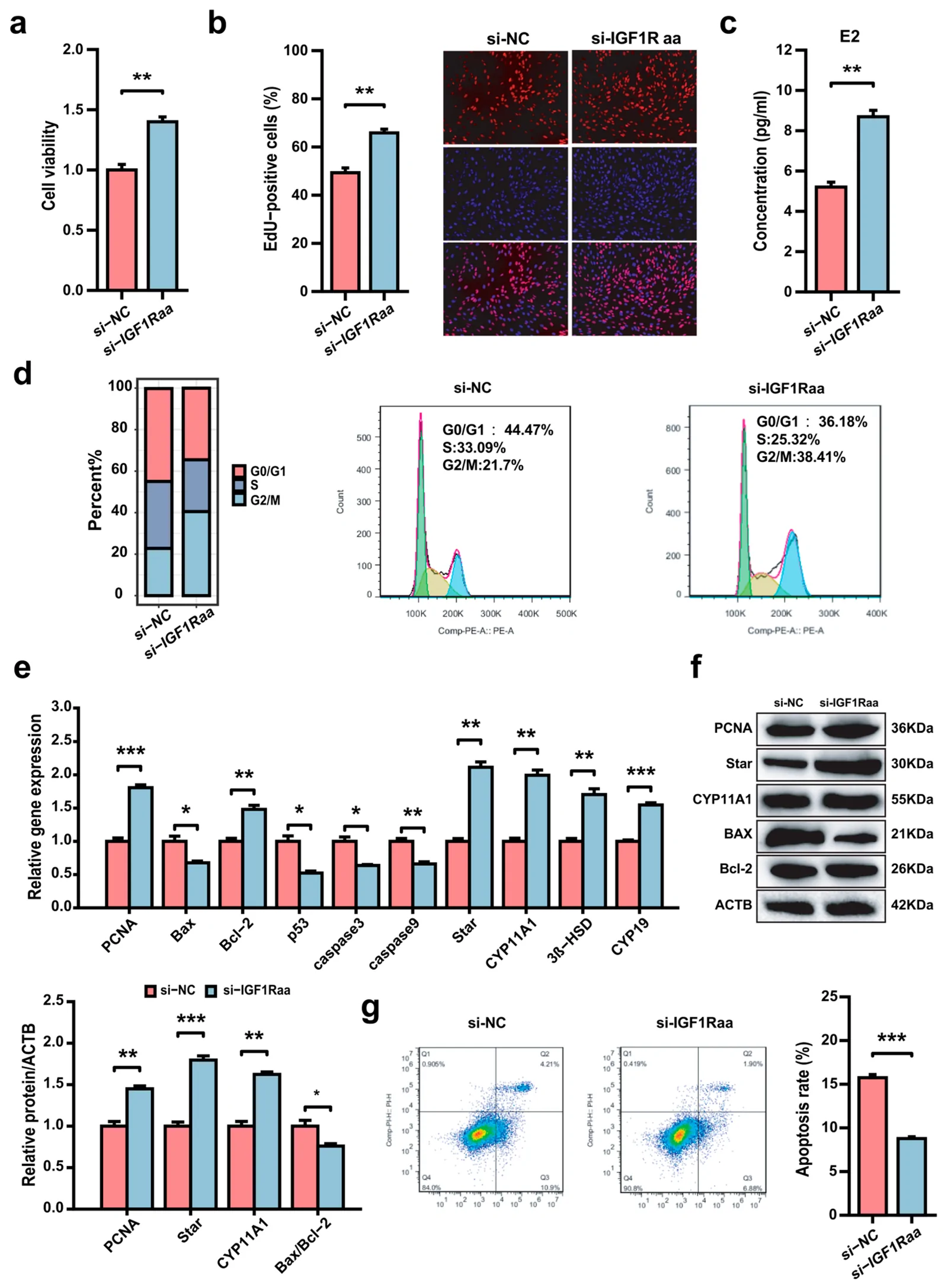
Effects of IGF1Raa knockdown on GC proliferation, apoptosis, and E2 secretion. (**a**) Cell viability of GCs after transfection with si-NC or si-IGF1Raa measured by CCK-8 assay. (**b**) Proliferation rate determined by EdU incorporation assay. (**c**) E2 concentration in culture supernatant measured by ELISA. (**d**) Cell cycle distribution analyzed by flow cytometry. (**e**) qRT-PCR analysis of mRNA expression of the proliferation marker PCNA, steroidogenic genes (Star, CYP11A1, 3β-HSD, CYP19), and apoptosis-related genes (p53, BAX, Bcl-2, Caspase3, Caspase9). (**f**) Western blot analysis of PCNA, STAR, CYP11A1, BAX, and Bcl-2 protein levels. (**g**) Apoptosis rate determined by flow cytometry. si-NC denotes the negative control siRNA group, and si-IGF1Raa denotes the siRNA targeting IGF1Raa group. * indicates *p* < 0.05, ** indicates *p* < 0.01 and *** indicates *p* < 0.001 (n = 3).

To assess the functional role of IGF1Raa in GCs, we designed specific siRNAs targeting IGF1Raa and transfected them into GCs. Knockdown of IGF1Raa resulted in a significant increase in cell viability (Figure 2a). Edu incorporation assays further revealed that the proliferation rate of si-IGF1Raa-treated cells was markedly higher than that of the si-NC group (Figure 2b), accompanied by notable alterations in cell cycle progression (Figure 2d). Consistently, both mRNA (Figure 2e) and protein levels (Figure 2f) of the proliferation marker PCNA were significantly upregulated.

ELISA measurements showed that E2 concentration in the culture medium was significantly elevated 48 h after IGF1Raa silencing (Figure 2c). Accordingly, the mRNA expression levels of steroidogenic genes Star, CYP11A1, 3β-HSD, and CYP19 were substantially increased (Figure 2e), and Western blot analysis confirmed that STAR and CYP11A1 protein levels were also upregulated (Figure 2f).

In contrast, apoptosis was enhanced upon IGF1Raa knockdown. Flow cytometry analysis demonstrated a significantly higher apoptotic rate in the si-IGF1Raa group compared with the si-NC group (Figure 2g). The mRNA levels of pro-apoptotic genes p53, BAX, Caspase3, and Caspase9 were elevated, whereas the anti-apoptotic gene Bcl-2 was downregulated (Figure 2e). Concordantly, BAX protein expression was increased and Bcl-2 protein expression was decreased, consistent with their respective mRNA changes (Figure 2f).

### 3.3. IGF1Raa Overexpression Suppresses GC Proliferation, Promotes Apoptosis, and Inhibits E2 Secretion In Vitro

**Figure 3:**
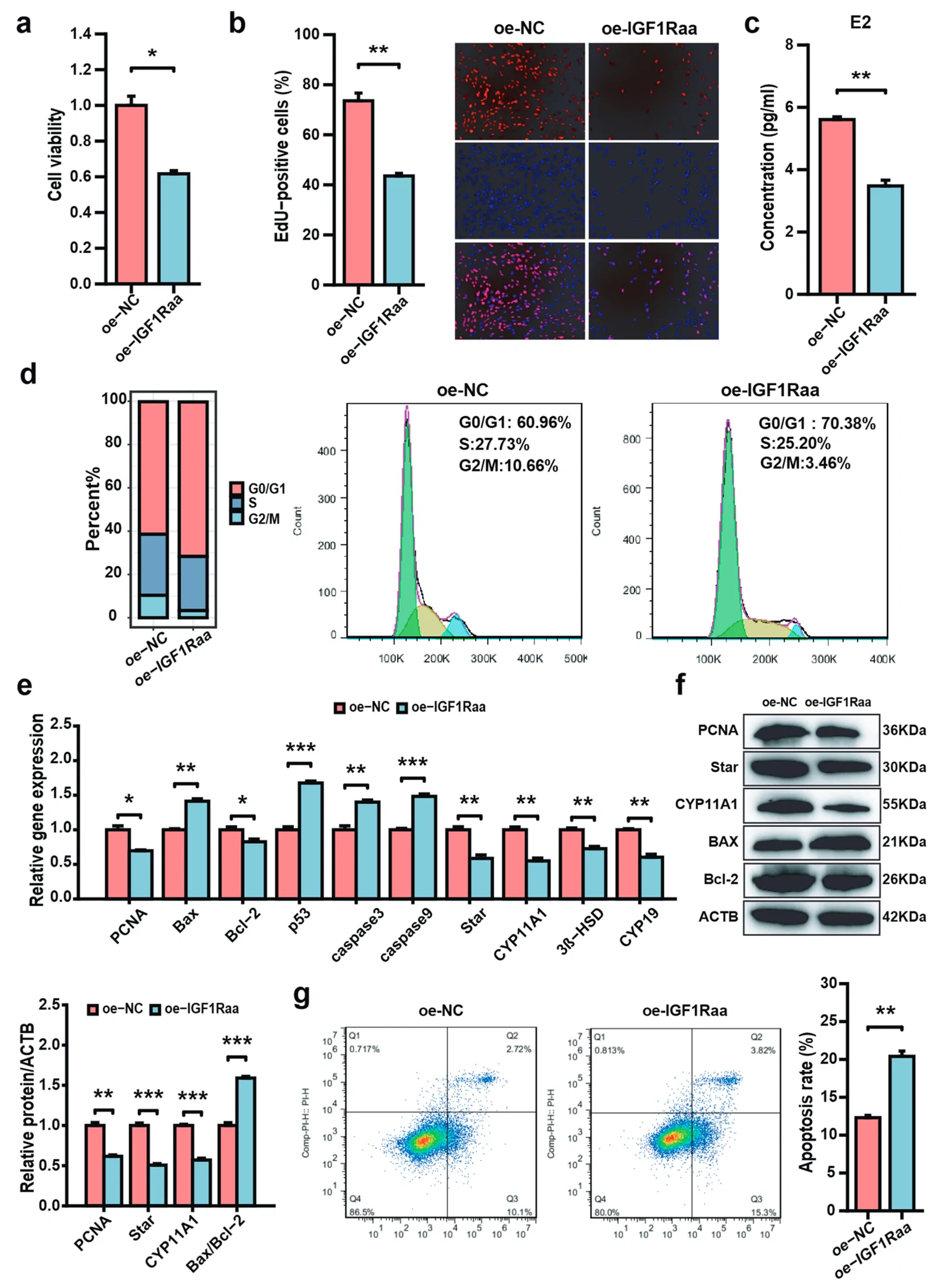
IGF1Raa overexpression suppresses GC proliferation, promotes apoptosis, and inhibits E2 secretion in vitro. (**a**) Cell viability of granulosa cells after transfection with oe-IGF1Raa or oe-NC measured by CCK-8 assay. (**b**) Proliferation rate determined by EdU incorporation assay. (**c**) E2 concentration in culture supernatant measured by ELISA. (**d**) Cell cycle distribution analyzed by flow cytometry. (**e**) qRT-PCR analysis of PCNA, steroidogenic genes, and apoptosis-related genes. (**f**) Western blot analysis of PCNA, STAR, CYP11A1, BAX, and Bcl-2 protein levels. (**g**) Apoptosis rate determined by flow cytometry. oe-NC represents the group transfected with an empty vector expressing only the tag protein, and oe-IGF1Raa represents the group overexpressing IGF1Raa. * indicates *p* < 0.05, ** indicates *p* < 0.01 and *** indicates *p* < 0.001 (n = 3).

Meanwhile, we also investigated the effects of IGF1Raa overexpression on the proliferation, apoptosis, and E2 synthesis of sheep GCs. Overexpression of IGF1Raa significantly reduced cell viability (Figure 3a) and suppressed the proliferation rate (Figure 3b), accompanied by a marked decrease in both mRNA (Figure 3e) and protein levels of PCNA (Figure 3f). Cell cycle progression was also correspondingly altered (Figure 3d).

Measurement of E2 levels revealed that IGF1Raa overexpression led to a significant reduction in E2 concentration in the culture medium (Figure 3c), along with decreased mRNA (Figure 3e) and protein expression (Figure 3f) of key steroidogenic enzymes. Flow cytometric analysis showed that the apoptotic rate was significantly higher in the oe-IGF1Raa group compared with the oe-NC group (Figure 3g). Accordingly, the mRNA levels of pro-apoptotic genes were upregulated (Figure 3e), BAX protein expression was increased, and Bcl-2 protein expression was decreased (Figure 3f). Collectively, these data demonstrate that overexpression of IGF1Raa inhibits GC proliferation, promotes apoptosis, and suppresses estrogen secretion.

### 3.4. IGF1Raa Functions Independently of IGF1R Expression in GCs

**Figure 4:**
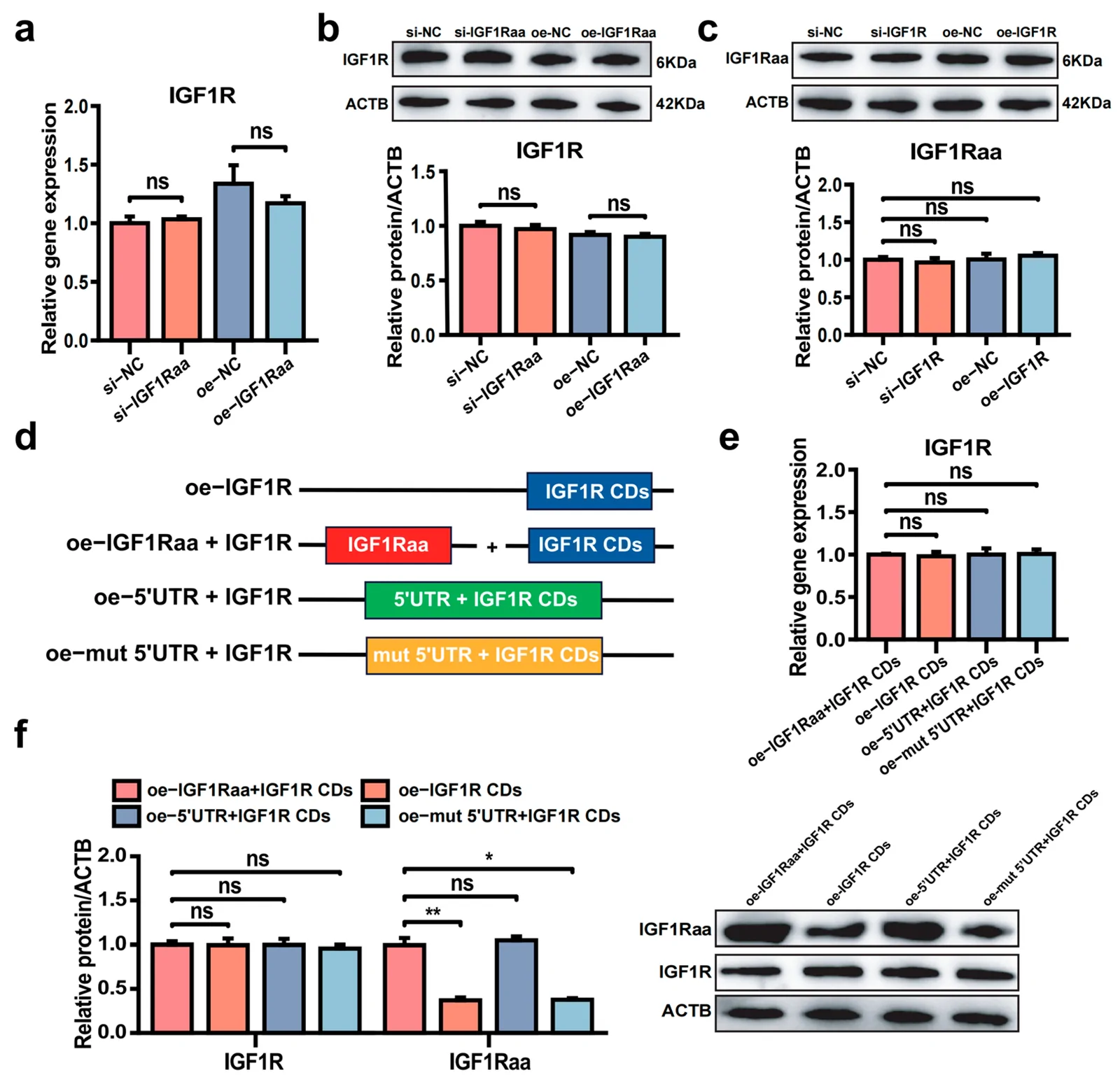
IGF1Raa functions independently of IGF1R expression in GCs. (**a**) qRT-PCR analysis of IGF1R mRNA levels after knockdown or overexpression of IGF1Raa. (**b**) Western blot analysis of IGF1R protein levels after knockdown or overexpression of IGF1Raa. (**c**) Western blot analysis of IGF1Raa protein levels after modulating IGF1R expression. (**d**) Schematic diagram of four constructs: oe-IGF1R CDS, oe-IGF1Raa + IGF1R CDS, oe-5′UTR + IGF1R CDS, and oe-mut 5′UTR + IGF1R CDS. (**e**) qRT-PCR analysis of IGF1R mRNA expression after transfection with the four constructs. (**f**) Western blot analysis or qRT-PCR analysis of IGF1R protein expression after transfection with the four constructs. si-NC represents the negative control group for siRNA knockdown, si-IGF1R represents GCs with IGF1R knockdown, and si-IGF1Raa represents GCs with IGF1Raa knockdown; oe-NC represents the negative control group for overexpression, oe-IGF1R represents GCs overexpressing IGF1R, and oe-IGF1Raa represents GCs overexpressing IGF1Raa. * indicates *p* < 0.05, ** indicates *p* < 0.01 (n = 3).

Given that sORF-IGF1Raa resides within the 5′UTR of the IGF1R mRNA, we asked whether IGF1Raa and IGF1R engage in reciprocal regulatory crosstalk. To test this, we manipulated IGF1Raa levels via both knockdown and overexpression and assessed IGF1R expression at the mRNA and protein levels. Neither manipulation altered IGF1R mRNA or protein abundance (Figure 4a,b). Conversely, modulating IGF1R expression likewise failed to change IGF1Raa protein levels (Figure 4c). To further rule out the possibility that the 5′UTR or the sORF competes for translational resources with the main CDS, we generated four distinct constructs: (i) IGF1R CDS alone (oe-IGF1R CDS), (ii) IGF1R CDS co-expressed with IGF1Raa (oe-IGF1Raa + IGF1R CDS), (iii) the full 5′UTR plus CDS (oe-5′UTR + IGF1R CDS), and (iv) the same 5′UTR + CDS backbone harboring a start-codon point mutation that abolishes IGF1Raa translation (oe-mut 5′UTR + IGF1R CDS) (Figure 4d). Strikingly, IGF1R mRNA (Figure 4e) and protein (Figure 4f) levels remained unchanged across all four groups. Collectively, these data establish that IGF1Raa functions as an autonomous peptide that operates independently of the canonical IGF1R coding sequence—neither perturbing nor being dictated by IGF1R expression.

### 3.5. Melatonin Regulates GC Function Partially Through Downregulation of IGF1Raa

**Figure 5:**
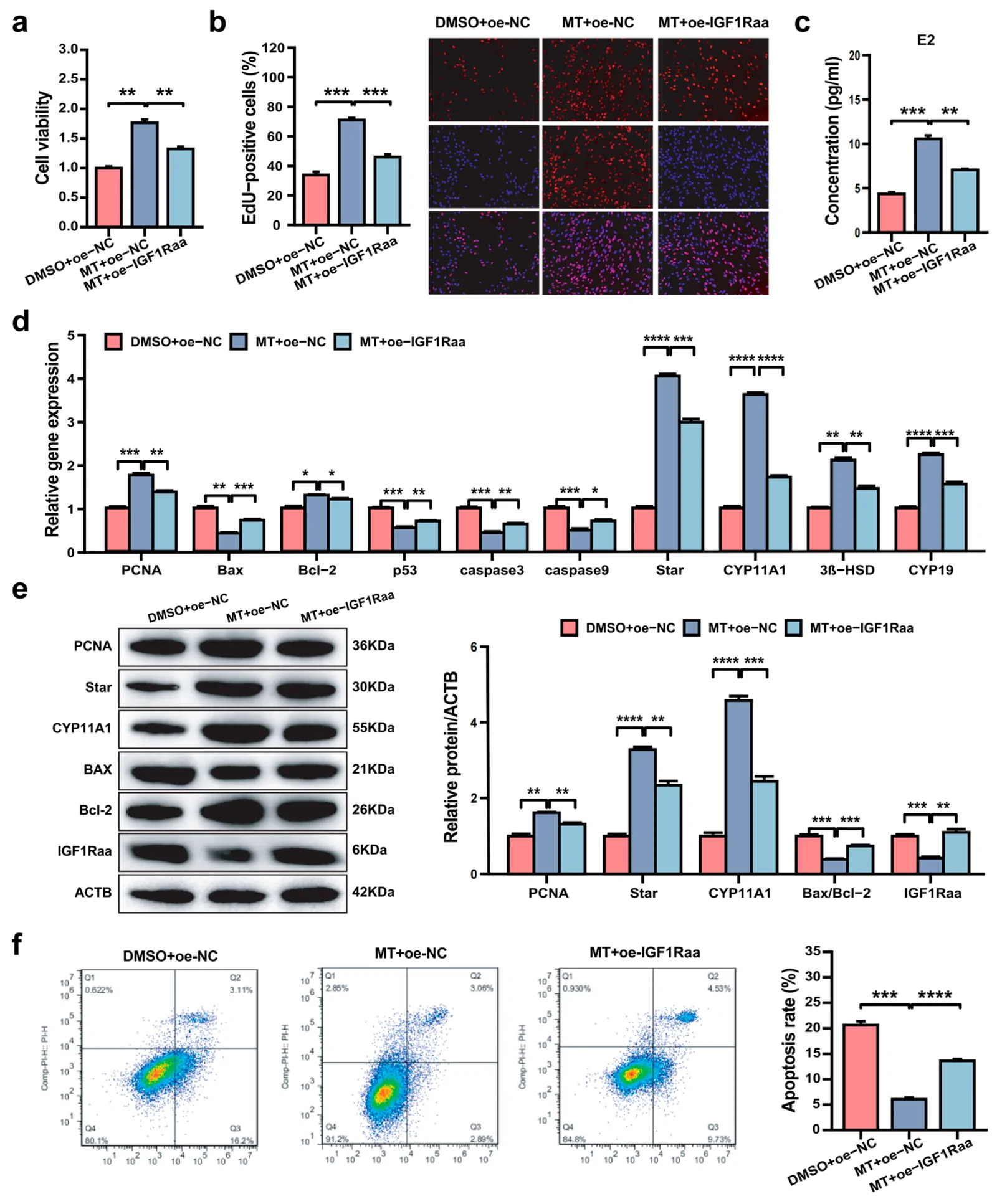
Melatonin regulates GC function partially through downregulation of IGF1Raa. (**a**) Cell viability of GCs in DMSO + oe-NC, MT + oe-NC, MT + oe-IGF1Raa by CCK8 assay. (**b**) Proliferation rate determined by EdU incorporation assay. (**c**) E2 concentration in culture supernatant measured by ELISA. (**d**) qRT-PCR analysis of PCNA, steroidogenic genes (Star, CYP11A1, 3β-HSD, CYP19), and apoptosis-related genes (p53, BAX, Bcl-2,Caspase3, Caspase9). (**e**) wb analysis of PCNA, STAR, CYP11A1, Bax/Bcl-2 and IGD1Raa protein expression. (**f**) Apoptosis rate determined by flow cytometry. DMSO represents the vehicle control with 0.1% DMSO, MT represents treatment with 10 nM melatonin, oe-NC represents transfection with the control overexpression plasmid, and oe-IGF1Raa represents transfection with the plasmid overexpressing IGF1Raa. * indicates *p* < 0.05, ** indicates *p* < 0.01, *** indicates *p* < 0.001 and **** indicates *p* < 0.0001 (n = 3).

Given that melatonin treatment significantly reduced IGF1Raa expression, we hypothesized that melatonin may exert its regulatory effects on GCs at least in part through suppressing IGF1Raa. To test this, we overexpressed IGF1Raa in GCs concurrently treated with melatonin. As expected, melatonin treatment alone significantly increased cell viability (Figure 5a), proliferation rate (Figure 5b), and E2 secretion (Figure 5c), accompanied by upregulation of PCNA, STAR, and other related genes at both the mRNA and protein levels (Figure 5d,e). Conversely, overexpression of IGF1Raa attenuated these melatonin-induced effects (Figure 5a–e). Furthermore, melatonin reduced the apoptosis rate in GCs, and this protective effect was partially abrogated by IGF1Raa overexpression (Figure 5f). These results indicate that melatonin promotes GC proliferation and steroidogenesis while inhibiting apoptosis, and that these effects are mediated, at least in part, through downregulation of IGF1Raa.

### 3.6. IGF1Raa Interacts with PPP2R2B in GCs

**Figure 6:**
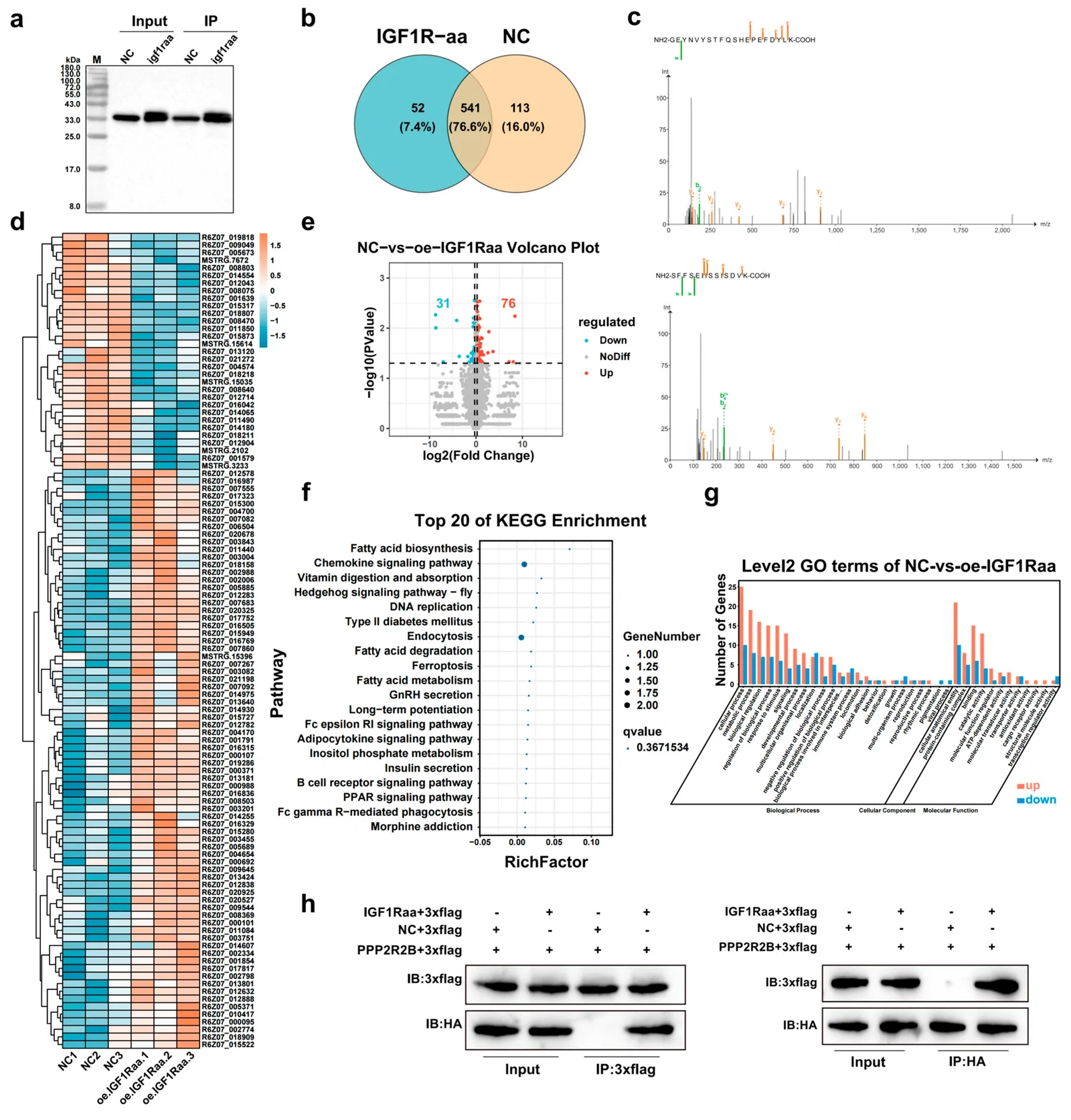
IGF1Raa interacts with PPP2R2B in GCs. (**a**) Western blot validation of Flag pull-down efficiency after Co-IP from cells overexpressing IGF1Raa-3 × Flag-GFP or control. (**b**) Venn diagram of proteins identified by mass spectrometry, showing proteins specifically associated with the oe-IGF1Raa group and those unique to the NC group. (**c**) Peptide information of PPP2R2B detected by mass spectrometry. (**d**) Heatmap of DEGs from RNA-seq of oe-IGF1Raa vs. control granulosa cells. (**e**) Volcano plot of DEGs. (**f**) KEGG pathway enrichment analysis showing significant enrichment of PPAR and other pathways. (**g**) GO functional enrichment analysis showing enrichment in cellular process and metabolic process terms. (**h**) Exogenous Co-IP validation: GCs co-transfected with IGF1Raa-3 × Flag and PPP2R2B-HA were subjected to immunoprecipitation with anti-Flag or anti-HA antibodies followed by Western blotting, confirming a physical interaction between IGF1Raa and PPP2R2B. NC represents transfection with the empty vector containing only 3 × Flag and GFP tags, and IGF1Raa represents transfection with the overexpression plasmid containing IGF1Raa, 3 × Flag, and GFP (n = 3).

To investigate the molecular mechanisms underlying IGF1Raa-mediated regulation of GC function, we performed Co-IP experiments using GCs overexpressing IGF1Raa-3 × Flag-GFP or 3 × Flag-GFP alone. Anti-Flag immunoprecipitation followed by Western blotting confirmed successful pull-down, as indicated by bands of the expected sizes in the IP fractions (Figure 6a). Mass spectrometry analysis of the co-precipitated proteins identified 52 proteins specifically associated with the oe-IGF1Raa group and 113 proteins unique to the NC group (Figure 6b). Using mass spectrometry, we found that PPP2R2B peptides co-precipitated with IGF1Raa (Figure 6c). To further explore the downstream effectors of IGF1Raa, we conducted RNA-seq on oe-IGF1Raa GCs. A total of 107 differentially expressed genes (DEGs) were identified (Figure 6d,e), which were significantly enriched in PPAR pathways (Figure 6f) and cellular process and metabolic process items (Figure 6g). Integrative analysis of the Co-IP and RNA-seq datasets highlighted PPP2R2B as a candidate interactor of IGF1Raa. Co-IP experiments confirmed that endogenous IGF1Raa associates with PPP2R2B. To validate this interaction, we co-transfected GCs with vectors encoding IGF1Raa-3 × Flag and PPP2R2B-HA. Reciprocal Co-IP using anti-Flag or anti-HA antibodies, followed by Western blotting, demonstrated that IGF1Raa and PPP2R2B physically interact (Figure 6h). These results establish PPP2R2B as a binding partner of IGF1Raa in GCs.

### 3.7. IGF1Raa Regulates the AMPK-mTORC1 Signaling Pathway via PP2A

PPP2R2B, a regulatory subunit of PP2A, has been shown to dephosphorylate AMPK at Thr172 and thereby suppress the AMPK/mTOR signaling cascade, ultimately modulating cell proliferation and apoptosis [24]. We therefore investigated whether IGF1Raa impinges on the PP2A/AMPK axis in GCs.

**Figure 7:**
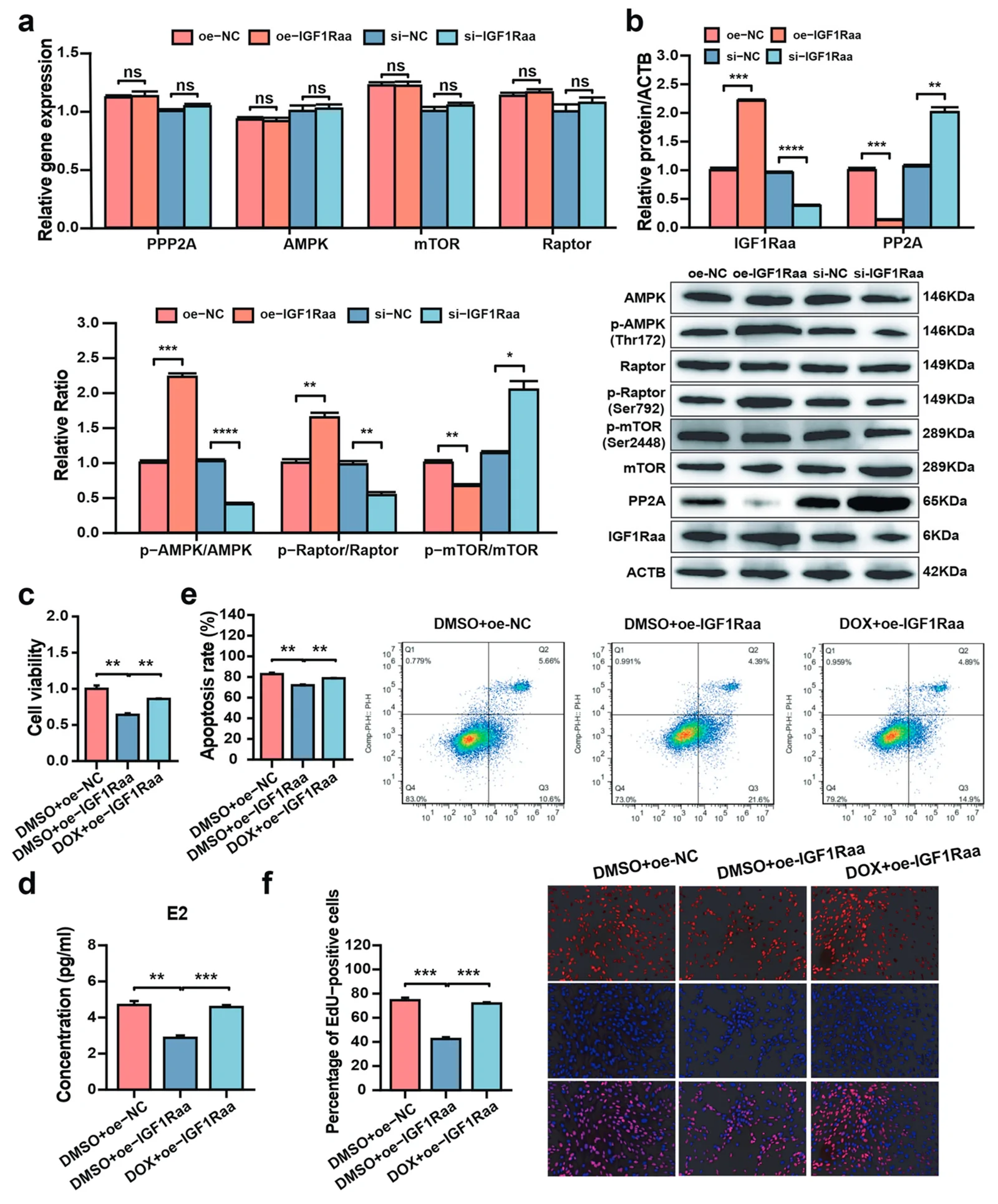
IGF1Raa regulates the AMPK-mTORC1 signaling pathway via PP2A. (**a**) qRT-PCR analysis of PPP2R2B, AMPK, mTOR, and Raptor mRNA levels after IGF1Raa knockdown or overexpression. (**b**) Western blot analysis of PPP2R2B protein, PP2A protein, and phosphorylation levels of AMPK at Thr172, Raptor at Ser792, and mTOR at Ser2448. (**c**) Cell viability of IGF1Raa-overexpressing cells treated with the AMPK inhibitor doxorubicin (DOX), measured by CCK-8 assay. (**d**) E2 secretion after DOX treatment measured by ELISA. (**e**) Apoptosis rate after DOX treatment determined by flow cytometry. (**f**) Proliferation rate after DOX treatment measured by EdU incorporation assay. si-NC represents the negative control group for siRNA knockdown, and si-IGF1Raa represents GCs with IGF1Raa knockdown; oe-NC represents the negative control group for overexpression, and oe-IGF1Raa represents GCs overexpressing IGF1Raa; DMSO represents the vehicle control with 0.1% DMSO, and DOX represents the group treated with 150 nM doxorubicin. * indicates *p* < 0.05, ** indicates *p* < 0.01, *** indicates *p* < 0.001 and **** indicates *p* < 0.0001 (n = 3).

Knockdown or overexpression of IGF1Raa did not affect PP2R2B mRNA levels (Figure 7a). However, silencing IGF1Raa markedly increased PP2A protein abundance, whereas IGF1Raa overexpression reduced it (Figure 7b). Notably, neither manipulation altered the total protein or mRNA levels of AMPK, mTOR, or Raptor (Figure 7a). In contrast, IGF1Raa knockdown significantly decreased phosphorylation of AMPK at Thr172 and Raptor at Ser792, while its overexpression exerted the opposite effect. Conversely, p-mTOR (Ser2448) exhibited an inverse phosphorylation pattern (Figure 7b).

To determine whether the AMPK pathway mediates IGF1Raa’s effects, we treated IGF1Raa-overexpressing GCs with the AMPK inhibitor doxorubicin (DOX). This pharmacological inhibition largely rescued the phenotypic defects induced by IGF1Raa overexpression: cell viability (Figure 7c), E2 secretion (Figure 7d), and proliferation rate (Figure 7f) were all restored, while the elevated apoptosis rate was reversed (Figure 7e).

In summary, these data demonstrate that IGF1Raa acts as a negative regulator of PP2A, thereby activating the AMPK/mTORC1 signaling cascade. Conversely, IGF1Raa knockdown relieves this suppression, leading to PP2A upregulation and pathway inactivation. Thus, IGF1Raa governs GC proliferation and apoptosis, at least in part, through modulation of the PP2A/AMPK/mTORC1 axis.

## 4. Discussion

In addition to regulating circadian rhythms, melatonin has also been shown to participate in reproductive regulation. Most melatonin in the body is secreted by the pineal gland [25]. In female animals, melatonin acts on the hypothalamus to regulate GnRH secretion, thereby influencing the hypothalamic–pituitary–ovaryl axis [26]. Meanwhile, it can also directly act on the ovary, affecting the intrafollicular environment, regulating follicular development, and modulating hormone secretion by GCs [27]. Micropeptides are defined as peptides consisting of fewer than 100 amino acids [16]. To date, studies on melatonin regulating granulosa cell function and follicular development through micropeptides remain limited. In our previous study, through Ribo-Seq and RNA-Seq profiling of melatonin-treated sheep GCs, we identified several melatonin-responsive sORFs with coding potential [23]. Among them, sORF6526 attracted our attention. The present study aimed to validate the coding capacity of sORF6526, investigate the biological functions of its encoded micropeptide in sheep GCs, and elucidate the mechanism of the melatonin–micropeptide–AMPK pathway.

In this study, we identified IGF1Raa, a 32-amino-acid micropeptide encoded by sORF6526 within the 5′UTR of the IGF1R mRNA, and elucidated its functional role in sheep GCs. Our findings establish a hierarchical signaling cascade with melatonin-IGF1Raa-PPP2R2B/PP2A-AMPK/mTORC1-GCs. This framework positions IGF1Raa as a nodal point that integrates melatonin signaling with the PP2A-AMPK axis, revealing a layer of post-transcriptional regulation operating independently of the canonical IGF1R receptor tyrosine kinase. Notably, this mechanism represents a shift in understanding how 5′UTR-encoded micropeptides can function as trans-acting bioactive molecules rather than merely cis-regulatory elements modulating downstream CDS translation.

Traditionally, 5′UTRs have been viewed primarily as cis-regulatory regions that modulate mRNA stability and translational efficiency [28]. However, advances in Ribo-seq have revealed that a subset of uORFs possess the capacity to encode functional micropeptides [29]. In our prior integrated Ribo-seq and RNA-seq profiling of melatonin-treated sheep GCs, we identified a previously unannotated small ORF within the 5′UTR of the IGF1R mRNA, which we designated sORF6526. In silico analyses using multiple computational tools consistently predicted that sORF6526 harbors coding potential. To experimentally validate this prediction, we constructed an overexpression vector in which sORF6526 was fused in-frame to a 3 × Flag-GFP tag. Transfection of this construct into GCs, followed by fluorescence microscopy and Western blot analysis, confirmed that sORF6526 is indeed translated into a detectable peptide product. Given its genomic localization within the IGF1R 5′UTR, we named the encoded peptide IGF1Raa. To enable detection of the endogenous protein, we generated a custom IGF1Raa-specific antibody. Western blot analysis not only verified the authentic expression of IGF1Raa in GCs but also revealed that melatonin treatment markedly reduced its protein levels. Furthermore, cross-species sequence alignment demonstrated that sORF6526 is highly conserved among human, mouse, and sheep, suggesting that IGF1Raa homologs likely exist across mammals and may carry evolutionarily conserved biological functions.

To further elucidate the functional role of IGF1Raa in GCs, we performed both knockdown and overexpression assays. The results demonstrated that IGF1Raa knockdown significantly promoted GC proliferation, apoptosis, and E2 secretion, accompanied by corresponding alterations in the mRNA and protein levels of related genes. In contrast, IGF1Raa overexpression exerted completely opposite effects. These findings collectively indicate that IGF1Raa plays a pivotal regulatory role in modulating GC functions.

Considering that IGF1Raa is located upstream of the IGF1R mRNA CDs, and given previous evidence that upstream open reading frames (uORFs) can influence the translational efficiency of the main CDS—thereby affecting canonical protein expression [30] and cellular function [31]—we next investigated whether IGF1Raa exerts its effects by regulating IGF1R expression. To address this, we performed reciprocal overexpression and knockdown experiments targeting both IGF1Raa and IGF1R. Our data revealed no direct mutual regulatory relationship between IGF1Raa and IGF1R, nor did disruption of the IGF1Raa start codon affect IGF1R expression. These findings challenge the classical view that uORFs primarily repress downstream CDS translation in a local manner [25]. Instead, they support an emerging concept in which certain uORF-encoded peptides act as independent bioactive molecules with distinct functions [32]. This distinction is important; whereas typical uORFs regulate downstream translation through their position, IGF1Raa appears to function via protein–protein interaction with PPP2R2B, a mode of action resembling conventional signaling peptides.

Melatonin is mainly secreted by the pineal gland in vivo, although studies have also shown that ovarian granulosa cells can synthesize small amounts of melatonin [33]. In female mammals, melatonin secreted by the pineal gland acts on the hypothalamus to regulate GnRH secretion, thereby influencing the function of the pituitary gland and ovaries [34]. Meanwhile, melatonin can also reach the ovaries via the bloodstream, enter the follicles, and regulate oocyte maturation and granulosa cell function [35]. Dark environments promote the secretion of melatonin from the pineal gland [6]. In short-day breeders such as sheep and deer, elevated melatonin levels stimulate hypothalamic GnRH secretion, thereby inducing estrus, while also facilitating follicular maturation and ovulation [36]. In our previous study, we found that in vitro melatonin supplementation enhanced the antioxidant capacity of sheep GCs and promoted E2 secretion [23]. Based on these observations, we sought to investigate the mechanism by which melatonin regulates sheep GCs. Here, we found that melatonin treatment significantly downregulated IGF1Raa expression. This observation led us to hypothesize that melatonin may regulate GC functions by targeting IGF1Raa. To test this, we treated GCs with melatonin while concurrently overexpressing IGF1Raa. The results demonstrated that IGF1Raa overexpression effectively attenuated the melatonin-induced effects on GC proliferation, apoptosis, and E2 secretion. These data provide compelling evidence supporting our hypothesis that melatonin modulates GC functions, at least in part, through the regulation of IGF1Raa.

To further elucidate the molecular mechanism underlying IGF1Raa function, we performed Co-IP coupled with MS using GCs overexpressing IGF1Raa or a 3 × Flag control. MS analysis identified PPP2R2B as a potential binding partner of IGF1Raa, which was subsequently confirmed by IP assays. Given that PPP2R2B serves as a regulatory subunit of PP2A, and PP2A is known to modulate the AMPK-mTOR signaling cascade [37] by regulating AMPK phosphorylation which is a central pathway governing cell proliferation, apoptosis [38]. Thus, we hypothesized that IGF1Raa may exert its effects on GCs by interacting with PPP2R2B to influence PP2A, thereby regulating the AMPK/mTOR axis. To test this hypothesis, we first examined the impact of IGF1Raa manipulation on the PP2A/AMPK pathway. Knockdown or overexpression of IGF1Raa did not significantly alter the mRNA levels of PP2A, AMPK, Raptor, or mTOR. However, IGF1Raa knockdown significantly upregulated the protein levels of PP2A and p-mTOR, while concomitantly downregulating p-AMPK and p-Raptor. These results indicate that IGF1Raa knockdown increases PP2A protein abundance, leading to the inactivation of the AMPK signaling pathway. Conversely, IGF1Raa overexpression exerted opposite effects on PP2A, p-AMPK, p-Raptor, and p-mTOR, further corroborating that IGF1Raa regulates the AMPK/mTOR pathway by modulating PP2A protein expression at the post-transcriptional level.

To definitively validate whether IGF1Raa affects GC functions through the AMPK pathway, we performed a rescue experiment by treating IGF1Raa-overexpressing GCs with DOX, a specific AMPK inhibitor. DOX treatment effectively attenuated the inhibitory effects of IGF1Raa overexpression on GC proliferation and E2 synthesis, and rescued the pro-apoptotic phenotype. These findings provide compelling evidence that IGF1Raa modulates GC proliferation, apoptosis, and estrogen secretion in an AMPK-dependent manner.

However, several limitations of this study should be acknowledged. First, all experiments were conducted in vitro using primary sheep GCs isolated from slaughterhouse ovaries. Although this system preserves the native cellular context, it cannot recapitulate the complex microenvironment of the intact follicle, including interactions with theca cells, oocytes, and the dynamic hormonal environment. Second, the functional consequences of IGF1Raa modulation on in vivo follicular development, ovulation rate, and fertility remain unexplored. Third, although this study demonstrated that melatonin can regulate IGF1Raa expression levels, the specific mechanism and the receptor through which melatonin exerts this regulation require further elucidation. Finally, given that sORF6526 is highly conserved but may exhibit tissue- or species-specific regulation, whether sORF6526 can encode micropeptides in other species and what biological functions they may possess await further investigation.

Taken together, our findings have direct practical implications for improving sheep reproductive performance. Melatonin supplementation has been widely employed in ovine reproductive management to promote follicular development and increase lambing rates [32,33]. In this study, we identified IGF1Raa as a downstream effector of melatonin, providing a potential molecular biomarker for evaluating the efficacy of melatonin-based reproductive therapies. Specifically, decreased IGF1Raa expression levels in GCs may serve as an indicator of favorable melatonin responsiveness. Furthermore, the high conservation of sORF6526 across mammals suggests that the IGF1Raa micropeptide may play a conserved role in regulating ovarian function beyond sheep, potentially offering valuable insights for reproductive regulation in other animals and even humans.

## 5. Conclusions

In summary, we identify IGF1Raa, a 32-amino-acid micropeptide encoded by a conserved sORF in the 5′UTR of the IGF1R mRNA, as a melatonin-responsive regulator of ovine GC function. IGF1Raa suppresses GC proliferation and E2 secretion while promoting apoptosis. Mechanistically, IGF1Raa binds PPP2R2B, reducing PP2A protein abundance and thereby sustaining p-AMPK and p-Raptor to activate the AMPK/mTORC1 cascade. IGF1Raa acts independently of the canonical IGF1R coding sequence, distinguishing it from classical uORF-mediated regulation. Melatonin downregulates IGF1Raa expression, and forced expression of IGF1Raa partially reverses melatonin’s pro-proliferative, pro-steroidogenic, and anti-apoptotic effects on GCs. This study reveals a novel mechanism by which melatonin regulates ovarian physiology through a 5′UTR-encoded micropeptide, expands the functional landscape of micropeptides in reproductive biology, and positions IGF1Raa as a potential target for melatonin-based therapeutic strategies aimed at improving fertility and treating ovarian dysfunction.

## Data Availability

The original contributions presented in this study are included in the article/Supplementary Material. Further inquiries can be directed to the corresponding author.

## Supplementary Materials

The following supporting information can be downloaded at: https://www.mdpi.com/article/10.3390/cells15191771/s1, Table S1: Primer sequences of genes; Table S2: Details of antibodies.

## Abbreviations

The following abbreviations are used in this manuscript:

GCs	granulosa cells
sORF	small open reading frame
UTR	untranslated region
E2	estradiol
Co-IP	co-immunoprecipitation
RNA-Seq	RNA sequencing
Ribo-Seq	ribosome profiling sequencing
CCK-8	Cell Counting Kit-8
EdU	EdU staining assay
Elisa	enzyme-linked immunosorbent assay
GnRH	gonadotropin-releasing hormone
ROS	reactive oxygen species (ROS)
CDS	coding sequences
FBS	fetal bovine serum
P/S	penicillin-streptomyci
DPBS	dulbecco’s phosphate-buffered saline
DOX	doxorubicin
DMSO	dimethyl sulfoxide
WB	Western blotting
MS	mass spectrometry
RNA-seq	RNA-sequencing
ANOVA	one-way analysis of variance
SEM	standard error of the mean
DEGs	differentially expressed genes
uORFs	upstream open reading frames
