# Supplementary material for: Melatonin Regulates Sheep Granulosa Cell Proliferation and Apoptosis Through a Novel Micropeptide, IGF1Raa
Source: Cells. 2026 Sep 29;15(19):1771. doi: 10.3390/cells15191771 (PMC13664153; doi:10.3390/cells15191771)
Supplement: Supplementary file 1 [file cells-15-01771-s001.zip › cells-4563375-supplementary.pdf]

Table S1. Primer sequences of genes

Primer name	Primer sequence	Product length/bp
PCNA	F: 5′-TGCAGATGTACCCCTTGTTGT-3′<br>R: 5′-CATCCTCGATCTTGGGAGCC-3′	83
Bax	F: 5′-GCATCCACCAAGAAGCTGAG-3′<br>R: 5′-CCGCCACTCGGAAAAAGAC-3′	130
Bcl-2	F: 5′-ATGTGTGTGGAGAGCGTCA-3′<br>R: 5′-AGAGACAGCCAGGAGAAATC-3′	182
p53	F:5′-TCAGATCCGTGGGCGTAAAC-3′<br>R:5′-AGTCTGAGTCAGGCCCTTCT-3′	191
caspase3	F:5′-TGAAACATGCCGCCTTCCTA-3′<br>R:5′-AGTGGCATACCCACATGACTG-3′	242
caspase9	F:5′-CCAGATGCCGTGTCTAGTCTG-3′<br>R:5′-ACAGTAAGGTAGGGTGAGGGG-3′	296
	F: 5′-CGCGACGTTTAAGCTGTGTG-3′	217

Star	R: 5′-ATAGGCAAGCTCTTGGTCGC-3′	
	F: 5′-TCCCCTTTCCACCAATCA-3′	
Cyp11A1	R: 5′-GCAGCGTTCACCCGTTTATT-3′	256
	F: 5′-ATCCACACCAGCACCATAG-3′	
3β-HSD	R: 5′-TTCCAGCACAGCCTTCTC-3′	144
	F: 5′-TTTTGCCGAGAAACGTGGTG-3′	
Cyp19A1	R: 5′-ATTGCCTCTTCAACCTGGGG-3′	133
	F: 5′-GTTCCTGGAGAAAGATGGCGA-3′	
AMPK	R: 5′- TTTGCCAACCTTCACTTTGCC-3′	122
	F: 5′-TAAGAAAACGGGGACCACGG-3′	
mTOR	R: 5′-CAAGAGAAGTGCTGTGGGCA-3′	282
	F: 5′- GGUUCCCGACUCGGCCGAGTT-3′	
si-IGF1Raa	R: 5′- CUCGGCCGAGUCGGGAACCTT-3′	

Table S2. Details of antibodies

Antibodies	Cat No.	Source	Dilution of WB
ACTB	bs-0061R	Bioss (Beijing, China)	1:5000
IGF1R	21707-1-AP	Proteintech(Wuhan, China)	1:1000
3xflag	**20543-1-AP**	Proteintech(Wuhan, China)	1:20000
PCNA	AF0239	Affinity (OH, USA)	1:1000
Star	bs3570R	Bioss (Beijing, China)	1: 5000
CYP11A1	bs1099R	Bioss (Beijing, China)	1:500
BAX	2772	CST(MA, USA)	1:1000
Bcl-2	3498	CST(MA, USA)	1:1000
PPP2A	13482-1-AP	Proteintech(Wuhan, China)	1:1000
AMPK	MCA2673GA	Bio-Rad, Hercules, CA, USA	1:500
mTOR	2983	CST (MA, USA)	1:1000
Raptor	DF7527	Affinity (OH, USA)	1:2000
p-mTOR(ser2448)	5536	CST (MA, USA)	1:1000
p-AMPK(thr172)	sc-33524	Santa Cruz, California, USA	1:1000
p-Raptor(ser792)	AF8350	Affinity (OH, USA)	1:2000
HRP-labeled Goat Anti-rabbit IgG(H+L)	31460	Invitrogen, Rockford, IL,USA	1:5000
HRP-labeled Goat Anti-Mouse IgG(H+L)	A0216	Beyotime, Haimen, China	1:1000
